# Review of the registration of clinical trials in UMIN-CTR from 2 June 2005 to 1 June 2010 - focus on Japan domestic, academic clinical trials

**DOI:** 10.1186/1745-6215-14-333

**Published:** 2013-10-14

**Authors:** Wentao Tang, Manabu Fukuzawa, Hirono Ishikawa, Kiichiro Tsutani, Takahiro Kiuchi

**Affiliations:** 1 Department of Drug Policy and Management, Graduate School of Pharmaceutical Sciences, The University of Tokyo, 7-3-1, Hongo, Bunkyo-ku, Tokyo 113-0033, Japan; 2 UMIN Center, The University of Tokyo Hospital, Tokyo, Japan; 3 Department of Health Communication, School of Public Health, The University of Tokyo, Tokyo, Japan

**Keywords:** UMIN-CTR, Clinical trial registration, Non-industry-funded trial, Accessibility, Comparative effectiveness research, Results publication

## Abstract

**Background:**

Established on 1 June 2005, the University Hospital Medical Information Network Clinical Trials Registry (UMIN-CTR) is the largest clinical trial registry in Japan, and joined the World Health Organization (WHO) registry network in October 2008. Our aim was to understand the registration trend and overall characteristics of Japan domestic, academic (non-industry-funded) clinical trials, which constitute the main body of registrations in UMIN-CTR. In addition, we aimed to investigate the accessibility of clinical trials in UMIN-CTR to people worldwide, as well as the accessibility of clinical trials conducted in Japan but registered abroad to Japanese people in the Japanese language.

**Methods:**

We obtained the data for registrations in UMIN-CTR from the UMIN Center, and extracted Japan domestic, academic clinical trials to analyze their registration trend and overall characteristics. We also investigated how many of the trials registered in UMIN-CTR could be accessed from the International Clinical Trials Registry Platform (ICTRP). Finally, we searched ClinicalTrials.gov for all clinical trials conducted in Japan and investigated how many of them were also registered in Japanese registries. All of the above analyses included clinical trials registered from 2 June 2005 to 1 June 2010.

**Results:**

During the period examined, the registration trend showed an obvious peak around September 2005 and rapid growth from April 2009. Of the registered trials, 46.4% adopted a single-arm design, 34.5% used an active control, only 10.9% were disclosed before trial commencement, and 90.0% did not publish any results. Overall, 3,063 of 3,064 clinical trials registered in UMIN-CTR could be accessed from ICTRP. Only 8.7% of all clinical trials conducted in Japan and registered in ClinicalTrials.gov were also registered in Japanese registries.

**Conclusions:**

The International Committee of Medical Journal Editors (ICMJE) announcements about clinical trial registration and the Ethical Guidelines for Clinical Research published by the Japanese government are considered to have promoted clinical trial registration in UMIN-CTR. However, problems associated with trial design, retrospective registration, and publication of trial results need to be addressed in future. Almost all clinical trials registered in UMIN-CTR are accessible to people worldwide through ICTRP. However, many trials conducted in Japan but registered abroad cannot be accessed from Japanese registries in Japanese.

## Background

Clinical trials are important for health professionals, researchers, patients, and governments because they provide high quality evidence for clinical practice. However, for various reasons, the actual situation of clinical trials is less than satisfactory. Publication bias caused by selective reporting of trials with positive results while withholding trials with negative/neutral results has been documented [1-3]. It is estimated that only half of all controlled trials have been reported since the first randomized controlled trial (RCT) in 1948 [4]. This will lead to biased decision-making in clinical practice, development of clinical practice guidelines, and resource allocation. Furthermore, patients participate in clinical trials as constituents of society, believing that they are contributing to medical knowledge for patients in the future. Hence, from an ethical perspective, trial results should be considered to be an important part of social property and given back to the public. Not reporting trial results will damage the trust between patients and investigators, as well as that between patients and research ethics review boards [4]. Moreover, problems associated with how to promote patient enrollment in clinical trials and how to prevent possible intended modification of the original protocol after the start of a trial remain to be solved.

To address the problems stated above, registration of clinical trials has been proposed as a possible solution since the 1960s [5]. The value and importance of an international registry of all clinical trials were further emphasized by RJ Simes in 1986 [6]. The objective of clinical trial registration is to make key information of all trials undertaken accessible to the public, thereby increasing the accountability and transparency of clinical trials, and reducing the chance of publication bias. In 2004, the scandal of concealment of safety problems in the clinical trial of paroxetine (Paxil; GlaxoSmithKline, Brentford, UK) aroused international concern and prompted the development of clinical trial registration [7,8]. In September 2004, the International Committee of Medical Journal Editors (ICMJE) announced their strategy to make the registration of clinical trials a prerequisite for consideration of publication [9]. One month later, an open meeting to discuss the international principles for trial registration was held in Ottawa, Canada. The resulting Ottawa statement detailed the requirement for registration of the protocols and results of all clinical trials [10].

In May 2005, the World Health Assembly endorsed a resolution (WHA 58.34) to establish a voluntary platform to link clinical trial registries to ensure a single point of access, as well as the unambiguous identification of trials, thereby enhancing access to information by patients, families, and patient groups [11]. This resolution led to the establishment of the International Clinical Trials Registry Platform (ICTRP) [12] by the World Health Organization (WHO) in 2007. The WHO also published a 20-item Trial Registration Data Set, which is considered to be the minimum standard for full registration [13]. Moreover, since its 2008 version, the Declaration of Helsinki by the World Medical Association requires every clinical trial to be registered in a publicly accessible database before recruitment of the first subject [14]. In addition, the Consolidated Standards of Reporting Trials (CONSORT) Statement, which has been adopted by most leading journals in the field of clinical trials, requires reporting of the registration number and name of the trial registry in item 23 [15].

In response to the global movement for clinical trial registration, and also to help provide information on clinical trials to Japanese nationals, the University Hospital Medical Information Network (UMIN) commenced the first Japanese clinical trials registry, UMIN-CTR [16], on 1 June 2005. UMIN was established in 1989 as a cooperative organization for national university hospitals in Japan. It is sponsored by the Ministry of Education, Culture, Sports, Science and Technology (MEXT) [17].

There are two other registries in Japan: the Japan Pharmaceutical Information Center Clinical Trials Information (Japic-CTI) [18] and the Japan Medical Association Center for Clinical Trials (JMACCT) [19]. In addition, an overall search portal for these three registries was established by the National Institute of Public Health (NIPH) in October 2007. The three registries and the search portal cooperate as a whole system and became a WHO-ICTRP Primary Registry (Japan Primary Registries Network, JPRN) in October 2008.

At present, the clinical trials conducted in Japan can be divided into two categories: 1) clinical trials regulated by Japanese Pharmaceutical Affairs Law (PAL) and Good Clinical Practice (GCP), covering trials for new drug application (called 'Chiken’ in Japanese) and post-marketing clinical trials sponsored by industry; and 2) other clinical trials that are not regulated by Japanese PAL and GCP. Most trials in this category are academic (non-industry-funded) clinical trials.

For category 1, the notifications of protocols of clinical trials for new drug application must be submitted to the Ministry of Health and Welfare (MHW, reorganized to be the Ministry of Health, Labour and Welfare (MHLW) since 2001) for review before trial start since October 1979 [20]. On the other hand, the notifications of protocols of post-marketing clinical trials sponsored by industry have been required to be submitted to MHW for review before trial start since December 1993 [21].

Although the annual number of notifications of clinical trial protocols submitted for regulation review is published on the Pharmaceuticals and Medical Devices Agency (PMDA) website [22], their contents are not accessible to public. At present, there is no legal document or ethical guideline to require the clinical trials in category 1 to be registered in a publicly accessible registry in Japan.

However, to increase clinical trial transparency for the benefit of patients and medical professionals, the International Federation of Pharmaceutical Manufacturers & Associations (IFPMA), the Japanese Pharmaceutical Manufacturers Association (JPMA), the Pharmaceutical Research and Manufacturers of America (PhRMA), and the European Federation of Pharmaceutical Industries and Associations (EFPIA) have approved the 'Joint Position on the Disclosure of Clinical Trial Information via Clinical Trial Registries and Databases’ in January 2005 [23]. This industrial joint position has been updated twice in November 2008 and November 2009. According to the latest version of this joint position (November 2009), member companies are required to register all their clinical trials in patients in a publicly accessible registry within 21 days of the start of patient enrollment [24].

Among more than 2,000 pharmaceutical companies in Japan, 70 companies joined JPMA by July 2013. Although the industrial joint position is neither a law nor a governmental guideline, it is considered to have constraint force to some extent on member companies, to promote the registration of clinical trials conducted by them.

For clinical trials in category 2 (trials not regulated by PAL or GCP), the Japanese government has taken the following measures to improve their registration: 1) since April 2007, MHLW has required registration in Japanese registries for clinical trials as a pre-condition for the application for Health and Labour Sciences Research Grants [25]; and 2) since April 2009, the MHLW has issued and applied the latest version of the Ethical Guidelines for Clinical Research in Japan. It was this version that first proposed the requirement for clinical trials conducted in Japan to be registered in Japanese registries [26]. Although the Ethical Guidelines for Clinical Research are only administrative guidelines without legal force, they are considered to be 'soft’ law and perceived as having effectiveness in regulating clinical research conducted in Japan [27].

We summarized the regulations on clinical trial information in Japan in Table 1, including the comparison with relevant regulations in the USA. In the USA, the registration of all private and public clinical trials that test effectiveness for 'serious or life-threatening’ conditions submitted to the Food and Drug Administration (FDA) under Investigational New Drug applications (INDs) was first mandated by the FDA Modernization Act, section 113 (FDAMA 113) in 1997 [28]. ClinicalTrials.gov [29], one of the most comprehensive registration platforms in the world, was established in 2000 as the result of this law. It accepts registrations of clinical trials all around the world and operates as a service of the US National Institutes of Health (NIH). Enacted since September 2007, Section 801 of the Food and Drug Administration Amendments Act (FDAAA 801) has expanded the scope for registration to be: interventional studies of drugs, biologics, and devices (whether or not approved for marketing); phase 2 through phase 4; at least one US site or IND or investigational device exemption (IDE) [30].

**Table 1:** Regulations on clinical trial information in Japan and USA

**Date**	**Japan**		**USA**	**Industrial position**^**c**^
	**Clinical trials regulated by PAL and GCP**	**Other clinical trials**		
October 1979	*Notification* of protocols of clinical trials for new drug application submitted to MHW (required by PAL)			
December 1993	*Notification* of protocols of post-marketing clinical trials submitted to MHW^a^			
November 1997			Registration of all trials that test effectiveness for 'serious or life-threatening’ conditions submitted to the FDA under INDs (required by FDAMA 113)	
January 2005				Registration of all trials except exploratory trials
April 2007		Registration of clinical trials with application for Health and Labour Sciences Research Grants^b^		
September 2007			Registration of interventional studies of drugs, biologics, and devices (whether or not approved for marketing); phase 2 through 4; at least one US site or IND or IDE (required by FDAAA 801)	
November 2008				Registration of all confirmatory clinical trials and all exploratory efficacy trials
April 2009		Registration of all trials conducted in Japan (required by Ethical Guidelines for Clinical Research)		
November 2009				Registration of all clinical trials in patients

^a^Required by Shin Iyakuhin Nado no Shiyo no Seiseki Nado ni Kansuru Cyosa Jisshi Keikakusyo ni tsu i te (the notification of protocol of use results surveys on new drugs) [21]; ^b^required by Heisei 19 Nendo Koseirodo Kagaku Kenkyuhi Hojyokin Kobo Yoko (requirement for the application of Health and Labour Sciences Research Grants) [25]; ^c^Joint Position on the Disclosure of Clinical Trial Information via Clinical Trial Registries and Databases (first version issued January 2005, updated November 2008 and November 2009, by IFPMA, JPMA, PhRMA, and EFPIA) [24]. EFPIA, European Federation of Pharmaceutical Industries and Associations; FDA, Food and Drug Administration; FDAAA, Food and Drug Administration Amendments Act; FDAMA, Food and Drug Administration Modernization Act; GCP, Good Clinical Practice; IDE, investigational device exemption; IFPMA, International Federation of Pharmaceutical Manufacturers & Associations; IND, Investigational New Drug application; JPMA, Japanese Pharmaceutical Manufacturers Association; MHW, Ministry of Health and Welfare; PAL, Pharmaceutical Affairs Law; PhRMA, Pharmaceutical Research and Manufacturers of America.

According to the results of our preliminary research, as of 1 June 2010, the total number of registrations (clinical trials and observational studies) in JPRN had reached 4,750 (UMIN-CTR: 3,595, 75.7%; Japic-CTI: 1,117, 23.5%; JMACCT: 38, 0.8%). Overall, 92.5% (3,324/3,595) of registrations in UMIN-CTR are academic (non-industry-funded) clinical studies, while 98.2% (3,530/3,595) of registrations in UMIN-CTR are domestic clinical studies (conducted only in Japan). Industry-funded studies are more likely to be registered in Japic-CTI, given that 94.0% (1,050/1,117) of registered studies in Japic-CTI are funded by industry. UMIN-CTR has been the main source of information for clinical studies registered in Japan, and the major body of the registrations in UMIN-CTR is constituted by Japan domestic, academic clinical studies.

More than 2,000 new registrations were received in UMIN-CTR in 2011, and the number of registrations is likely to continue to increase in the future. However, no systematic review of the registered clinical trials in UMIN-CTR has been conducted. To understand the registration trend and overall characteristics of the clinical trials registered in UMIN-CTR, and to provide insightful advice for the future improvement of Japanese clinical trials, we conducted a retrospective analysis of the clinical trials registered in UMIN-CTR in the first 5 years since its establishment (from 2 June 2005 to 1 June 2010). The focus of our analyses was on Japan domestic, academic clinical trials because these studies constitute the major body of all the registrations in UMIN-CTR, and may have considerably different trial characteristics from industry-funded or global studies. To reflect the main characteristics of the clinical trials registered in UMIN-CTR more precisely, we finally defined the object of our analyses as Japan domestic, academic clinical trials registered in UMIN-CTR in the first 5 years since its inception.

Moreover, since UMIN-CTR was incorporated into JPRN as a part of the primary registry of ICTRP at 3 years after its establishment, the aspect of whether the clinical trials in UMIN-CTR, especially those trials registered before its incorporation into ICTRP, can be accessed successfully through ICTRP became our concern. Since this issue has not previously been investigated, we analyzed the accessibility of the clinical trials in UMIN-CTR to people around the world through ICTRP. In addition, some clinical trials conducted in Japan are registered in foreign registries, and the aspect of whether such trials are also registered in Japanese registries in the Japanese language remains unclear. Therefore, we analyzed the accessibility of clinical trials conducted in Japan but registered abroad to Japanese people in the Japanese language.

Overall, our research involved four research questions. First, how many Japan domestic, academic clinical trials were registered each month in the first 5 years since the establishment of UMIN-CTR? Second, what are the overall characteristics of the registered clinical trials? Third, what is the accessibility of trials registered in UMIN-CTR to people around the world through ICTRP? Fourth, what is the accessibility of trials conducted in Japan (with at least one listed site in Japan) but registered abroad to Japanese people in the Japanese language?

## Methods

### Registry

UMIN-CTR is a web-based registration platform that accepts the registration of all types of clinical trials and observational studies. According to the UMIN-CTR’s principle, if Japan is included in the countries of recruitment for a study, registration in both English and Japanese is required. Otherwise, registration in English only is acceptable. One UMIN unique trial number (study ID) is generated automatically for the identification of each registered trial [31].

Based on the WHO 20-item Trial Registration Data Set and the requirement for registration information proposed by the ICMJE, UMIN-CTR has added some items according to local needs to form a set of 85 registration items [31]. These 85 items reflect the information for a clinical trial on five domains [32]: basic information; objective; design; administration information; and progress. Approximately 53 of these items must be registered (this number may vary according to individual trials). Most of the registered information will be accessible to the public from the date of disclosure, which can be specified by the registrant. The registration and search for clinical trial information are free.

With respect to the reporting of trial results in a clinical trials registry, FDAAA 801 has mandated the submission of summary result data for certain trials of drugs, biologics, and devices to ClinicalTrials.gov (generally not later than 1 year after the completion date) since September 2008 [30]. ClinicalTrials.gov was expanded in 2008 to establish a results database for reporting trial results. On the other hand, reporting of trial results in UMIN-CTR is optional, and no results database has yet been established. Although the aspect of whether or not the trial results have been published must be chosen when registering a trial, the URL for published results and detailed trial results in text form are only optional items and are not mandated to be registered [31].

### Data and screening

We obtained the full data containing all the registered information of the registrations in UMIN-CTR from 2 June 2005 to 1 June 2010. We obtained these data from the UMIN Center (downloaded on 18 January 2011), and imported them into an Excel file for screening.

For research questions 1 and 2, we conducted three-step screening to extract trials that were eligible for analyses. First, we analyzed the region/country of the registered studies and extracted the Japan domestic studies. Next, we analyzed the funding sources of the extracted studies and excluded industry-funded studies. Finally, observational studies were excluded and clinical trials were obtained for further analyses.

For research question 3, we used the full data for the registrations in UMIN-CTR from 2 June 2005 to 1 June 2010 for analyses. For research question 4, we chose ClinicalTrials.gov as the object for analysis. We searched ClinicalTrials.gov by specifying 'Japan’ in the 'country’ field, and '06/02/2005 to 06/01/2010’ in the 'first received’ field in the advanced search, to identify studies conducted in Japan and registered during the analysis period. We downloaded the information (20 items) for the identified registrations into an Excel file and then excluded observational studies to obtain clinical trials for analyses.

### Analysis methods

#### Research question 1: Monthly registration numbers of clinical trials

The monthly numbers of registrations from 2 June 2005 to 1 June 2010 were plotted along a time axis to observe the registration trend. The date of disclosure of trial information was taken as the registration date. This analysis was conducted from 11 April 2011 to 25 July 2011.

#### Research question 2: Overall characteristics of registered trials

The information for registered items was examined by categorized analyses to understand the overall characteristics of the registered clinical trials. The methods for categorization were based on the registration form of UMIN-CTR. According to the local needs for information by Japanese clinicians and researchers, 15 items were analyzed with primary concern and are discussed in this article. Another 13 items were analyzed with secondary concern and the results of these items will not be discussed in this article (only presented in an additional file for reference). The items of primary concern and secondary concern are listed in Table 2. This analysis was conducted from 11 April 2011 to 25 July 2011.

**Table 2:** Items of primary concern and secondary concern for analyses

**Primary concern**	**Secondary concern**
1. Malignancy/other	1. Objective
2. Genomic information	2. Developmental phase
3. Confirmatory/exploratory	3. Randomization unit
4. Explanatory/pragmatic	4. Stratification
5. Basic design	5. Dynamic allocation
6. Randomization	6. Institution consideration in allocation
7. Blinding	7. Concealment
8. Control	8. Blocking in allocation
9. Number of arms	9. Purpose of intervention
10. Type of intervention	10. Lower age limit
11. Target sample size	11. Sex
12. Trial period	12. Primary sponsor
13. Publication of results	13. Funding source
14. Disclosure of trial before/after trial start	
15. Disease classification by specialty	

#### Research question 3: Accessibility of trials registered in UMIN-CTR through ICTRP

To study the accessibility of clinical trials registered in UMIN-CTR to people around the world using ICTRP, we estimated how many clinical trials registered in UMIN-CTR from 2 June 2005 to 1 June 2010 could be accessed from ICTRP. ICTRP is not a clinical trials registry. It provides a searchable database containing trial registration datasets that are made available by data providers and primary registries around the world, including JPRN.

We first investigated the number of clinical trials and observational studies among all the registrations in UMIN-CTR over 5 years (from 2 June 2005 to 1 June 2010). Two types of study IDs (one beginning with 'C00000’, which was used in the early stage, and the other one beginning with 'UMIN’, which is currently used) were assigned for these studies and the following search of ICTRP was based on these study IDs. Hence, we first divided all the studies into two categories according to their ID type, and then investigated the number of clinical trials and observational studies in each category.

Subsequently, we investigated how many of these studies could be accessed from ICTRP using three steps. First, we searched ICTRP using 'JPRN-UMIN*’ and 'JPRN-C00000*’ in the basic search. 'JPRN’ is used in ICTRP to denote study records imported from Japanese registries. The two identifiable strings in the study IDs ('C00000’ and 'UMIN’) denote that a study is registered in UMIN-CTR. Therefore, study records imported from UMIN-CTR should have main IDs beginning with 'JPRN-UMIN’ or 'JPRN-C00000’. All studies registered should be assigned a study ID containing an identifiable string representing the registry, meaning that we could use the identifiable strings in IDs to search for studies registered in specific registries. The identifiable strings for several registries used in our research are shown in Table 3.

**Table 3:** Clinical trial registries and identifiable strings in study IDs

**Clinical trial registry**	**Identifiable string in study ID**
UMIN-CTR	'UMIN’, 'C00000’
Japic-CTI	'Japic’
JMACCT	'JMA’
ClinicalTrials.gov	'NCT0’

Japic-CTI, Japan Pharmaceutical Information Center Clinical Trials Information; JMACCT, Japan Medical Association Center for Clinical Trials; UMIN-CTR, University Hospital Medical Information Network Clinical Trials Registry.

Second, we noticed that some studies had multiple records in ICTRP, which were imported from different registries because they were registered not only in UMIN-CTR, but also in other registries. For some of these studies, the records imported from other registries were taken as the main records, while the records from UMIN-CTR were the secondary records. Since secondary records cannot be searched on ICTRP, some studies registered not only in UMIN-CTR, but also in other registries may not be found in step 1. However, the main records for such studies may contain the secondary IDs assigned by UMIN-CTR. Hence, for the studies that were registered in UMIN-CTR during the 5-year period but not found in step 1, we searched for their study IDs assigned by UMIN-CTR in the 'secondary ID’ field in the advanced search on ICTRP to examine whether or not they could be accessed.

Third, it was found that the records of some studies imported from UMIN-CTR were not taken as the main records, and that their study IDs assigned by UMIN-CTR were also not contained in the corresponding main records. Such studies cannot be found in either step 1 or step 2. Hence, for the studies that were registered in UMIN-CTR during the 5-year period but not found in step 1 or step 2, we searched for them in a basic search of ICTRP using their secondary IDs or titles registered in UMIN-CTR as the search terms. For example, if study 'C000000063’ was not found in step 1 or step 2, its secondary ID registered in UMIN-CTR, 'NCT00131027’, was searched for in ICTRP to see whether or not it could be accessed.

Since this analysis was only focused on clinical trials without observational studies, the studies that were registered in UMIN-CTR, but not found on ICTRP, by the three-step search described above were checked to examine whether or not they were clinical trials. Subsequently, the number of clinical trials that were registered in UMIN-CTR in the 5-year period and accessible from ICTRP was calculated.

#### Research question 4: Accessibility of trials conducted in Japan (with at least one listed site in Japan) but registered abroad to Japanese people in the Japanese language

We searched for clinical studies conducted in Japan (with at least one listed site in Japan) and registered from 2 June 2005 to 1 June 2010 (first received date) in ClinicalTrials.gov. We then downloaded the relevant information (20 items) for the identified registrations into an Excel file on 29 November 2012. We extracted the clinical trials from these registrations and tried to assess how many of them were also registered in Japanese registries and accessible in the Japanese language.

If a trial was also registered in Japanese registries, an identifiable string ('C00000’ , 'UMIN’ , 'Japic’ , or 'JMA’) should be found in the 'other IDs’ item of that trial in the downloaded data. Hence, we used the four identifiable strings to detect trials that were also registered in Japanese registries.

We also noticed some trials that were first registered in ClinicalTrials.gov and then registered in Japanese registries. Such registrations in Japanese registries may not be reported to ClinicalTrials.gov, and thus the IDs of these trials issued by Japanese registries may be missing in the data downloaded from ClinicalTrials.gov. However, the records of such trials in Japanese registries should contain the IDs issued by ClinicalTrials.gov because study IDs previously issued by other registries must be registered in Japanese registries. Therefore, we also searched UMIN-CTR, Japic-CTI, and JMACCT for complementation. We used the term 'NCT0’ (an identifiable string in the study IDs of ClinicalTrials.gov) to search for UMIN-CTR in the 'UMIN’s unique trial number or other study IDs’ field, Japic-CTI in the 'full text search’ field, and JMACCT in the 'free word’ field. We identified trials that were registered in Japanese registries and ClinicalTrials.gov during the period of the analysis.

By summing up the results of such a double-sided search and excluding duplicate records of the same trial, we identified trials that were registered in Japanese registries and ClinicalTrials.gov among all the trials conducted in Japan and registered in ClinicalTrials.gov from 2 June 2005 to 1 June 2010. We also analyzed the type of funding source of these trials by categorizing them as 'industry-funded’ , 'others-funded’ and 'industry + others-funded’. The rates of registration in Japanese registries among clinical trials conducted in Japan and registered in ClinicalTrials.gov were calculated in each category and compared by a two-sided Fisher’s exact test (α = 0.05). All the analyses were conducted using Microsoft Excel 2007 (Microsoft, Seattle, WA, USA) and SPSS version 15.0 (SPSS, Chicago, IL, USA).

## Results

### Full data for 3,595 registrations in UMIN-CTR from 2 June 2005 to 1 June 2010

**Figure 1:**
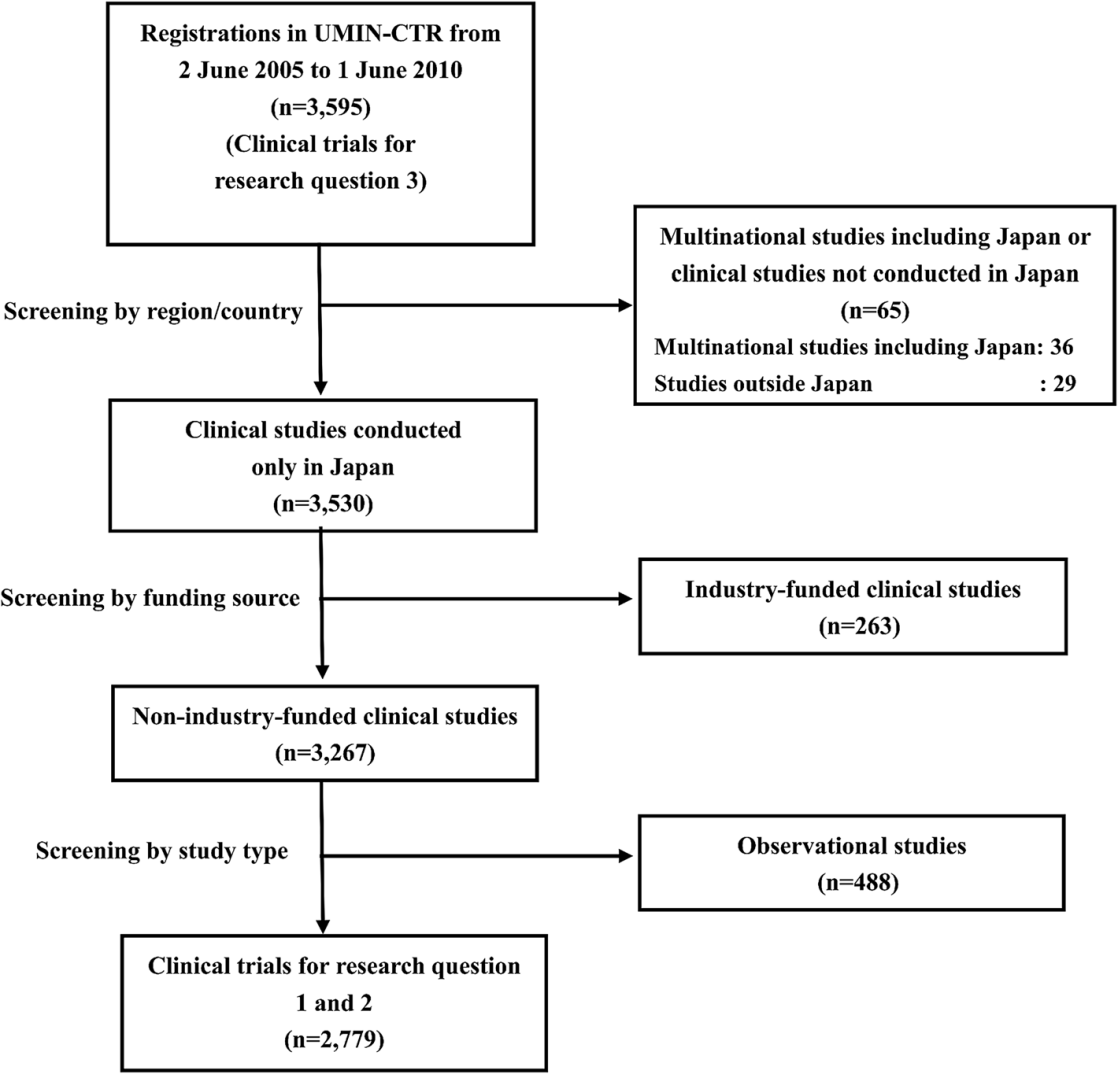
Screening for research questions 1 and 2.

For research questions 1 and 2, three-step screening was undertaken. First, 3,530 Japan domestic studies were extracted. Subsequently, we excluded industry-funded trials and obtained 3,267 studies. Finally, 488 observational studies were excluded and 2,779 clinical trials were identified (Figure 1). For research question 3, all 3,595 registrations in the full dataset were included for analyses. For research question 4, a total of 1,519 studies conducted in Japan and registered in ClinicalTrials.gov from 2 June 2005 to 1 June 2010 were identified. We excluded 110 observational studies and obtained 1,409 clinical trials.

### Research question 1: Monthly registration numbers of clinical trials

**Figure 2:**
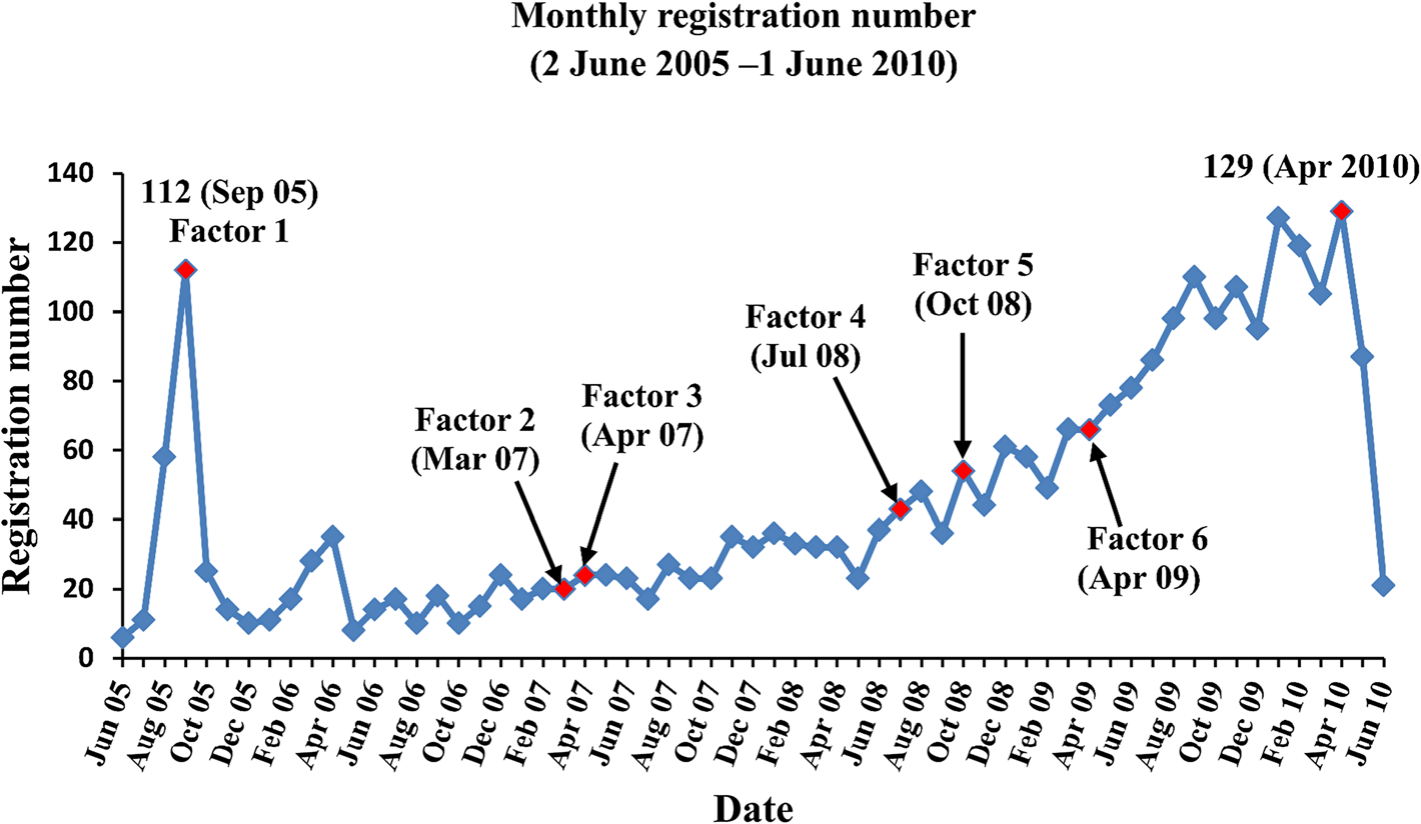
**Registration trend of clinical trials in UMIN-CTR.** The total registration number from 2 June 2005 to 1 June 2010 is 2,779. Factor 1: ICMJE announcement about clinical trial registration (September 2005) [9]. Factor 2: New 5-Year Clinical Trial Activation Plan (March 2007) [34]. Factor 3: Requirement for application for Health and Labour Sciences Research Grants (April 2007) [25]. Factor 4: Revised ICMJE announcement about clinical trial registration (July 2008) [33]. Factor 5: Formation of JPRN as an ICTRP Primary Registry (October 2008) [17]. Factor 6: Ethical Guidelines for Clinical Research (April 2009) [26]. ICMJE, International Committee of Medical Journal Editors; ICTRP, International Clinical Trials Registry Platform; JPRN, Japan Primary Registries Network; UMIN-CTR, University Hospital Medical Information Network Clinical Trials Registry.

The monthly registration numbers in UMIN-CTR from 2 June 2005 to 1 June 2010 are shown in Figure 2. A peak of registration was observed in September 2005 (n = 112). After the peak, the monthly registration remained at <40 from October 2005 to May 2008. From June 2008, this pattern began to change and the number of registrations started to increase with fluctuations. From April 2009, the registration entered a period of steady and rapid growth. One year later, the monthly registration reached 129, which was the highest record since the start of UMIN-CTR. The number of registrations in June 2010 was only 21 because we only included the trials registered on the first day of that month.

### Research question 2: Overall characteristics of registered trials

The results of the analysis of items of primary concern are summarized in Table 4. Trials focusing on malignancy accounted for nearly half of all trials (47.8%). Only 8.3% of trials used genomic information while they were being carried out. With regard to the trial characteristics, the prevalence of confirmatory/exploratory trials was 36.3% versus 27.4%, whereas that of explanatory/pragmatic trials was 11.7% versus 38.9%. Since these two items did not have to be registered, appreciable data were missing.

**Table 4:** Items of primary concern

**Category**	**Number of trials**	**%**
**1**. **Malignancy**/**others**		
Malignancy	1,327	47.8
Others	1,452	52.2
**2**. **Genomic information**		
Used	230	8.3
Not used	2,549	91.7
**3**. **Confirmatory**/**exploratory**		
Confirmatory	1,010	36.3
Exploratory	762	27.4
Missing data	1,007	36.2
**4**. **Explanatory**/**pragmatic**		
Explanatory	326	11.7
Pragmatic	1,081	38.9
Missing data	1,372	49.4
**5**. **Basic design**		
Parallel	1,334	48.0
Crossover	135	4.9
Factorial	17	0.6
Single-arm	1,289	46.4
Expanded access	4	0.1
**6**. **Randomization**		
Randomized	1,346	48.4
Non-randomized	1,433	51.6
**7**. **Blinding**		
Open	2,181	78.5
Assessor blinded	298	10.7
Single-blind (participants)	87	3.1
Single-blind (investigators and assessors)	33	1.2
Double-blind	180	6.5
**8**. **Control**		
Placebo	187	6.7
No treatment	249	9.0
Active	959	34.5
Dose comparison	70	2.5
Historical	281	10.1
Uncontrolled	1,033	37.2
**9**. **Number of arms**		
1	1,289	46.4
2	1,282	46.1
3	145	5.2
>**4**	63	2.3
**10**. **Type of intervention**^**a**^		
Medicine	2,074	74.6
Vaccine	112	4.0
Genetic	16	0.6
Food	75	2.7
Device, equipment	273	9.8
Behavior, custom	133	4.8
Maneuver	445	16.0
Others	0	0.0
**11**. **Target sample size**		
1 to 10	210	7.6
11 to 100	1,781	64.1
101 to 1,000	725	26.1
1001 to 1,0000	58	2.1
>10,000	5	0.2
**12**. **Trial period** (**months**)^**b**^		
0 to 6	138	5.0
7 to 12	180	6.5
13 to 36	1,057	38.0
37 to 60	611	22.0
>60	428	15.4
Missing data	365	13.1
**13**. **Report of results**^**c**^		
Unpublished	2,501	90.0
Published	136	4.9
Partially published	142	5.1
**14**. **Disclosure of trial before**/**after trial start**^**d**^		
Disclosure first	303	10.9
Trial start first	2,476	89.1
**Total**	2,779	100

^a^Values within this category are not mutually exclusive (total number: >2,779); ^b^period between trial start date and date of last patient’s final visit; ^c^'published’: the final trial results were published regardless of the kind of publication form (journals, academic conferences, webpages, and so on), 'partially published’: only the results of interim analyses were published; ^d^date of first enrollment was taken as the trial start date.

Parallel comparisons (48.0%) and single arms (46.4%) were the designs most often used in all trials. There were more non-randomized trials (51.6%) than randomized trials (48.4%). Most trials were open without any blinding (78.5%) or only the assessors were blinded (10.7%). Only 6.5% of trials had a double-blind design. A total of 88.6% (1,269/1,433) of non-randomized trials and 56.9% (1,242/2,181) of unblinded trials were single-arm designs. Only 5.2% of trials were conducted with three arms.

'Medicine’ was the most studied type of intervention (74.6%) and 'maneuver’ was also studied in many registered trials (16.0%). The target sample sizes of trials were concentrated in the categories of 11 to 100 (64.1%) and 101 to 1,000 (26.1%). Trials with a sample size of >1,000 accounted for only 2.3% of all trials. Nearly 90% of all trials were carried out for >12 months (13 to 36 months, 38.0%; 37 to 60 months, 22.0%; >60 months, 15.4%) (Table 4).

**Figure 3:**
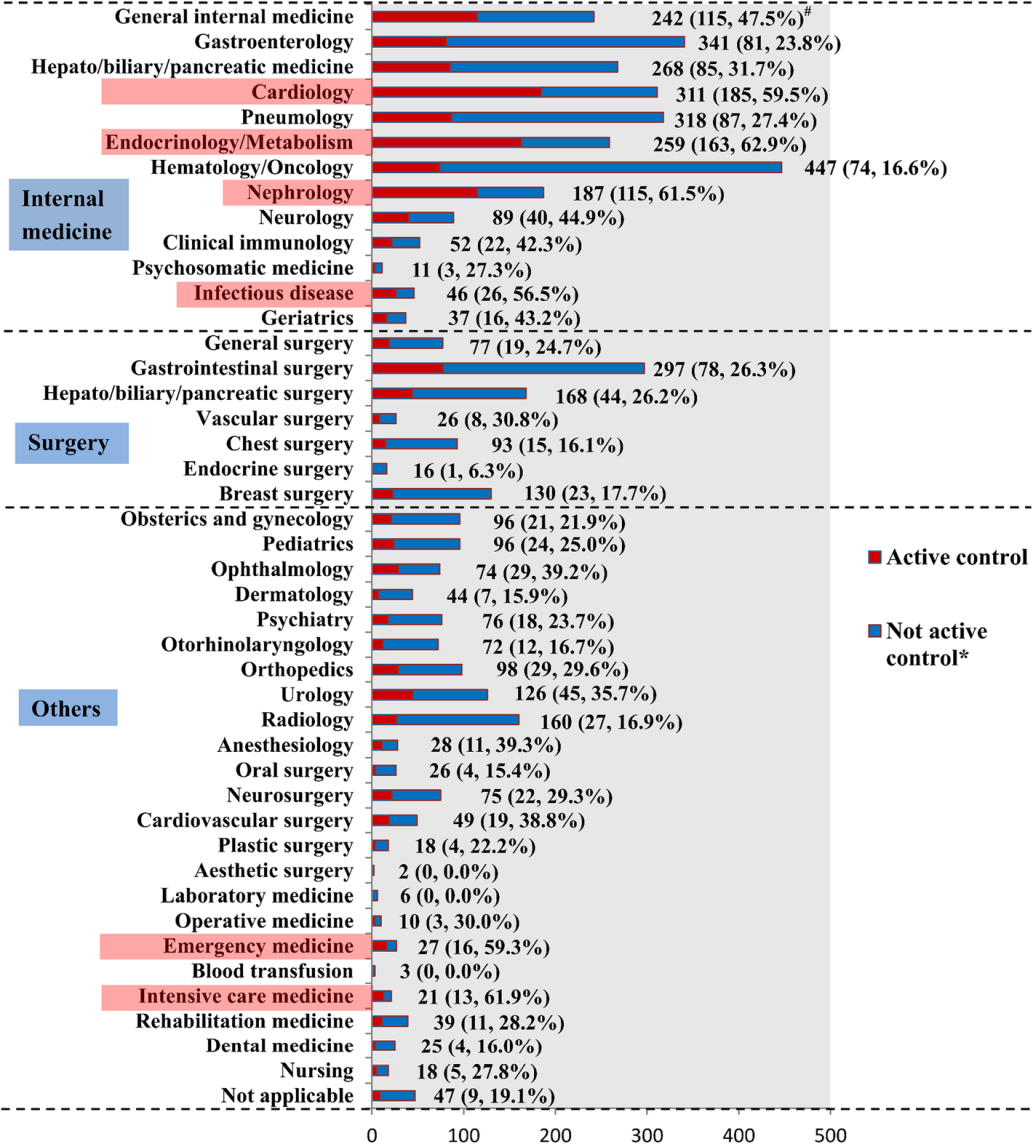
**Disease-classified registration numbers by specialty and control type.** The total number of registrations is 2,779. The registration numbers in different subcategories are not mutually exclusive. Six subcategories with a prevalence of <50% for using an active control are highlighted in pink. ^#^All data labels: total number (number of active controls, percentage of active controls). *No active control: placebo, no treatment, dose comparison, historical, uncontrolled.

The prevalence of publication and partial publication of trial results in the registry were low (publication, 4.9%; partial publication, 5.1%). Moreover, only 10.9% of all trials disclosed the trial information before trial commencement. The numbers of registered studies and active control studies classified by disease specialty are presented in Figure 3. Hematology/oncology was the most studied field (n = 447). Many clinical trials (>300) were based on gastroenterology, cardiology, and pneumology. Fewer than 10 trials were identified in aesthetic surgery, laboratory medicine, and blood transfusion. The prevalence of using an active control in clinical trials exceeded 50% in 6 of 44 fields (ranked from high to low: endocrinology/metabolism, intensive care medicine, nephrology, cardiology, emergency medicine, and infectious disease). These fields are highlighted in pink in Figure 3. A prevalence of <10% for using an active control was observed in four fields (endocrine surgery, blood transfusion, laboratory medicine, and aesthetic surgery). The overall prevalence for use of an active control in clinical trials in UMIN-CTR was 34.5% (Table 4). Clinical trials conducted without a control were found to account for a large proportion of all registered trials (37.2%).

The results of the analysis of items of secondary concern are shown in an additional file (see Additional file 1).

### Research question 3: Accessibility of trials registered in UMIN-CTR through ICTRP

**Figure 4:**
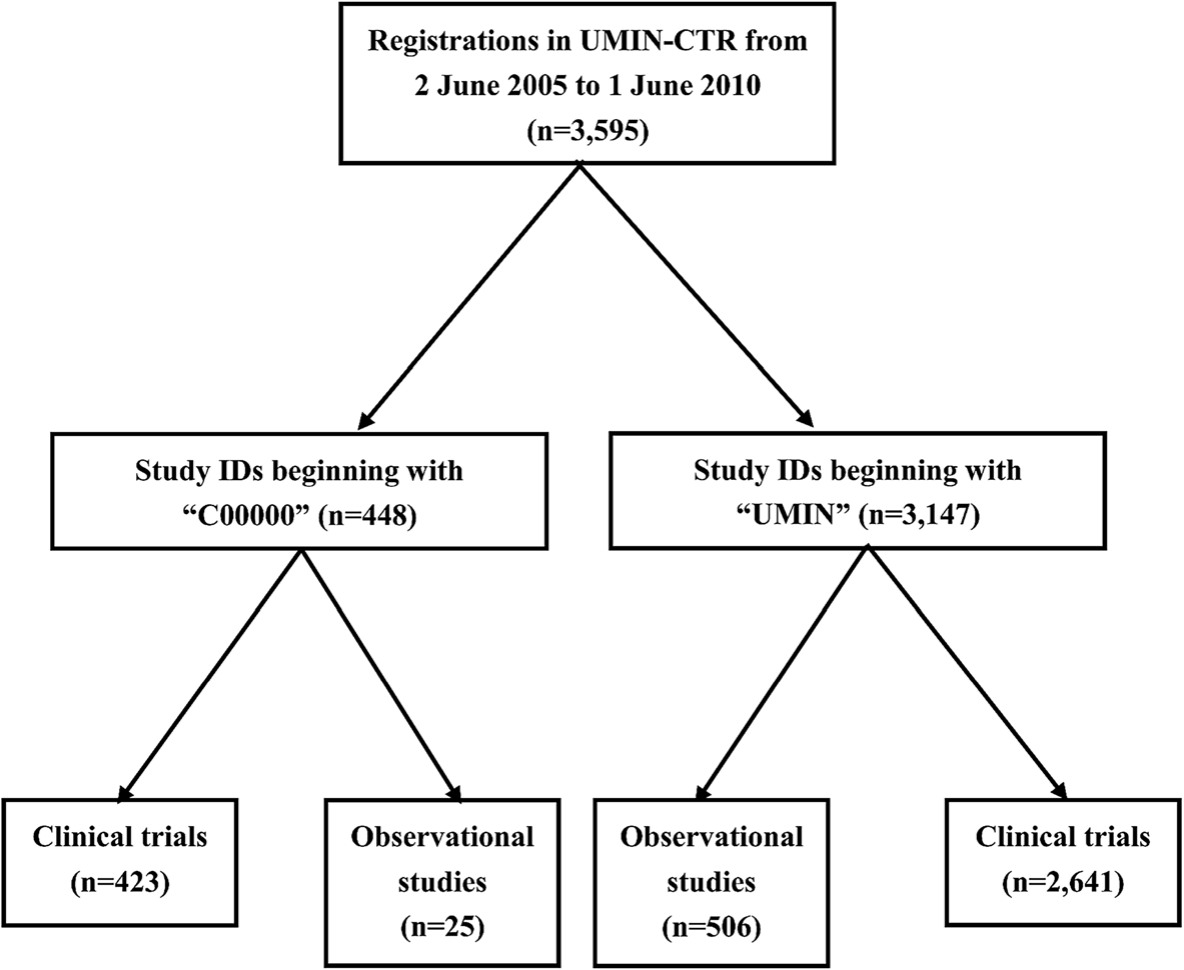
Numbers of registrations classified by study ID and study type.

The numbers of registrations classified according to study ID type and study type are presented in Figure 4. Among 3,595 registrations in UMIN-CTR from 2 June 2005 to 1 June 2010, 448 studies had a study ID beginning with 'C00000’ , while the study IDs of the remaining 3,147 studies began with 'UMIN’. Among the 448 studies beginning with 'C00000’, 423 were clinical trials and 25 were observational studies. With regard to the 3,147 studies beginning with 'UMIN’, 2,641 were clinical trials and 506 were observational studies. The total number of clinical trials registered in UMIN-CTR during the 5-year period was 3,064.

All 3,147 studies beginning with 'UMIN’ were found in the three-step search of ICTRP (step 1: 3,118; step 2: 11; step 3: 18). Apart from study 'C000000060’, all the other 447 studies beginning with 'C00000’ were found in the three-step search of ICTRP (step 1: 430; step 2: 8; step 3: 9). Study 'C000000060’ is a clinical trial, so all 3,064 clinical trials except for one were accessible from ICTRP.

### Research question 4: Accessibility of trials conducted in Japan (with at least one listed site in Japan) but registered abroad by Japanese people in the Japanese language

Based on a search using downloaded data from ClinicalTrials.gov, we identified 96 trials that were registered in Japanese registries and ClinicalTrials.gov (UMIN-CTR, 50; Japic-CTI, 45; JMACCT, 0; UMIN-CTR + Japic-CTI, 1). Based on a complementary search of UMIN-CTR, Japic-CTI, and JMACCT, we identified a further 27 trials that were also registered in Japanese registries and ClinicalTrials.gov (UMIN-CTR, 23; Japic-CTI, 4; JMACCT, 0). Therefore, 123 trials were found to be registered in Japanese registries and ClinicalTrials.gov. Such trials accounted for 8.7% (123/1,409) of all trials conducted in Japan and registered in ClinicalTrials.gov from 2 June 2005 to 1 June 2010 (Table 5).

**Table 5:** Clinical trials conducted in Japan and registered in ClinicalTrials.gov (2 June 2005 to 1 June 2010)

**Clinical trial**	**Trials only registered in ClinicalTrials.gov**	**Trials registered in Japanese registries and ClinicalTrials.gov**				**Total**
		**UMIN**	**Japic**	**UMIN**/**Japic**^**b**^	**Subtotal**	
Industry-funded	975	2	49	1	52	1,027
	(94.9%)	(0.2%)	(4.8%)	(0.1%)	(5.1%)	(100.0%)
Others-funded	285	67	0	0	67	352
	(81.0%)	(19.0%)	(0.0%)	(0.0%)	(19.0%)	(100.0%)
Industry + others funded	23	4	0	0	4	27
	(85.2%)	(14.8%)	(0.0%)	(0.0%)	(14.8%)	(100.0%)
Unknown funding source^a^	3	0	0	0	0	3
	(100.0%)	(0.0%)	(0.0%)	(0.0%)	(0.0%)	(100.0%)
**Total**	1,286	73	49	1	123	1,409
	(91.3%)	(5.2%)	(3.5%)	(0.1%)	(8.7%)	(100.0%)

Number (percentage calculated with numbers in the last column). ^a^Recorded as 'intervention’; ^b^registered in UMIN-CTR and Japic-CTI. Japic-CTI, Japan Pharmaceutical Information Center Clinical Trials Information; UMIN-CTR, University Hospital Medical Information Network Clinical Trials Registry.

According to the funder type, all 1,409 trials could be classified as follows: industry-funded, 1,027; others-funded, 352; industry + others-funded, 27; unknown funder, 3. We compared the prevalence of registration in Japanese registries in the three categories (industry-funded; others-funded; industry + others-funded) by Fisher’s exact test. We found a significant difference between these three categories (*P* <0.001). Industry-funded trials had the lowest prevalence of registration in Japanese registries (5.1%), while others-funded trials had the highest prevalence (19.0%) (Table 5).

Among 1,286 trials that were only registered in ClinicalTrials.gov, 975 (75.8%) trials were industry-funded, 285 (22.2%) were others-funded, and 23 (1.8%) were industry + others-funded.

## Discussion

### Research question 1: Monthly registration numbers of clinical trials

The curve of the monthly registration numbers in UMIN-CTR can be divided into four stages: 1) peak (around September 2005); 2) stationary (October 2005 to May 2008); 3) slow increase with fluctuations (June 2008 to March 2009); and 4) rapid and steady increase (April 2009 to April 2010).

To explain the observed peak around September 2005 in stage 1, the ICMJE announcement published in 2004 is considered to be the most important factor. In this announcement, ICMJE member journals required the registration of a clinical trial in a public registry before enrollment of patients as a condition of consideration for publication [9]. This policy was applied from 1 July 2005. For trials that began enrollment before this date, ICMJE member journals required registration by 13 September 2005. Considering that publication is an important motivation for initiating clinical trials, conditions for publication may have substantial effects on registration behaviors. A similar peak around September 2005 was also observed in the analysis of ClinicalTrials.gov conducted in 2005 by Zarin *et al*. [28].

However, the monthly registrations obviously decreased after this peak and remained at <40 for approximately 2.5 years (stage 2). The requirement for clinical trials to be registered in Japanese registries as a pre-condition for applications for Health and Labour Sciences Research Grants from April 2007 did not show any obvious effect on the registration trend.

The situation changed from June 2008 when the monthly registration curve entered a stage of slow increase with fluctuations (stage 3). In 2007, the ICMJE revised their announcement about clinical trial registration and expanded the scope of registration to include all clinical trials involving humans [33], whereas the announcement in 2004 did not require the registration of trials studying pharmacokinetics and major toxicity. The revised version was applied from July 2008, and could have been one possible factor for the increase in monthly registrations. Other factors that could have promoted the increase in registration were the New 5-Year Clinical Trial Activation Plan issued by the Japanese government (MEXT/MHLW) in March 2007 [34] and the formation of JPRN as an ICTRP Primary Registry in October 2008.

From April 2009, it can be observed from Figure 2 that the curve started to increase rapidly and steadily (stage 4). The Ethical Guidelines for Clinical Research issued in April 2009 may have exerted an important positive effect on the registration of clinical trials in Japan.

### Research question 2: Overall characteristics of registered trials

We compared the results of analyses of UMIN-CTR with reports from similar studies: 1) study on ICTRP by Viergever and Ghersi in 2011 (including data registered from 17 June 2008 to 17 June 2009 in nine registries around the world) [35]; and 2) study on ClinicalTrials.gov by Califf *et al*. in 2012 (including data in the fields of oncology, cardiovascular, and mental health, registered from October 2007 to September 2010) [36]. Such studies reflect the global situation to some extent.

Some obvious differences in the characteristics of registered clinical trials between UMIN-CTR, ICTRP, and ClinicalTrials.gov were found. With regard to the basic design, 46.4% of trials in UMIN-CTR adopted a single arm without a control group. This prevalence was obviously higher than those reported for ICTRP (162/731, 22.2%) and ClinicalTrials.gov (12,144/40,970, 29.6%). Although a single-arm design is frequently used in phase I and phase II trials, and especially in oncology settings, an appropriate control group is considered a key point for obtaining rigorous and informative evidence from clinical trials. Using a historical control from other studies for comparison may be feasible in single-arm trials, but the validity of results from such comparisons cannot be considered to be high, as different studies may vary quite a lot in their study design and other aspects. The reasons for the comparatively high prevalence of single-arm designs among the trials registered in UMIN-CTR require further exploration.

Single-arm designs need neither randomization nor blinding, and may be carried out with fewer participants. Therefore, the high prevalence of single-arm designs also influenced these characteristics of the registered trials. We found that a large part of the non-randomized and unblinded trials followed a single-arm design. The prevalence of randomized trials in UMIN-CTR was 48.4%, being lower than those in ICTRP (518/731, 70.9%) and ClinicalTrials.gov (27,027/40,970, 66.0%). The prevalence of unblinded trials was higher in UMIN-CTR than in ClinicalTrials.gov (78.5% versus 54.2%), while the prevalence of double-blind trials was lower in UMIN-CTR than in ClinicalTrials.gov (6.5% versus 32.2%). Moreover, the prevalence of using three arms in clinical trials, such as intervention/active control/placebo, was very low (5.2%) in UMIN-CTR. When analyzing the target sample size, it was found that the trials in ClinicalTrials.gov tended to enroll more participants (101 to 1,000: 33.8%; >1,000: 3.9%) than the trials in UMIN-CTR (101 to 1,000: 26.1%; >1,000: 2.3%).

To explain the above differences in the trial characteristics between UMIN-CTR and ICTRP/ClinicalTrials.gov, it should be considered that all industry-funded trials were excluded from our analysis of UMIN-CTR, but were included with a prevalence of 44.0% in the analyses of both ICTRP and ClinicalTrials.gov. Moreover, the object and period for the analysis of UMIN-CTR were not completely identical with those for ICTRP and ClinicalTrials.gov. However, the differences in the trial characteristics between UMIN-CTR and ICTRP/ClinicalTrials.gov may imply the need for improvement in the design of clinical trials in Japan.

In recent years, comparative effectiveness research (CER) has been drawing increasing attention from patients, clinicians, policymakers, and healthcare payers for its expected role in supporting evidence-based decision-making in clinical practice and health policy [37,38]. CER is the conduct and synthesis of systematic research comparing different interventions and strategies to prevent, diagnose, treat, and monitor health conditions. Considering that one of the key characteristics of CER is the direct comparison of effective interventions [37] (except for some diseases without effective intervention in current practice), most CER originates from studies using an active control. According to our analysis of UMIN-CTR, 34.5% of trials adopted an active control. This prevalence is higher than that reported by another study analyzing trials conducted in the USA on 15 specific research areas and registered in ClinicalTrials.gov from January 2007 to April 2010 (22.3%) [39].

With regard to this difference in the prevalence of active control use between our analysis of UMIN-CTR and that of ClincalTrials.gov, the different research design wherein industry-funded trials were excluded from our analysis but included in the analysis of ClinicalTrials.gov should be considered. In addition, the analysis of ClinicalTrials.gov examined trials conducted in only 15 specific research areas whereas no such specification was made in our research. Besides the differences in research design, two other factors may lead to the comparatively high prevalence of active control use found in our analysis. The first is the strong interest in CER by Japanese investigators of academic clinical trials. The second is the different perception of active/placebo control and preferences between Japanese and American investigators and patients. More detailed studies concerning these two factors are expected to promote the future development of CER.

Pragmatic trials are considered to be better aligned with CER than explanatory trials because of their features, such as using more active controls instead of placebo, including broader study populations, and assessing more patient-centered outcomes [40]. Apart from 1,372 missing data, we discovered that 1,081/1,407 (76.8%) of the trials registered in UMIN-CTR were labeled as 'pragmatic trials’. However, the overall prevalence of using an active control was far lower (34.5%), even in addition to trials with a 'no treatment’ control (9.0%), which can study conditions without an available active control. Actually, in UMIN-CTR, the registration of pragmatic/explanatory trials is based on the registrant’s judgment and we are not sure whether the statistics reflect the true situation. There is no absolute boundary between pragmatic trials and explanatory trials. Pragmatic features and explanatory features may exist simultaneously in different dimensions of one trial [41]. Sometimes judging whether a trial is pragmatic or explanatory is difficult. Moreover, although many trials that adopted a single-arm design may be labeled as 'pragmatic’ because of their pragmatic features in certain dimensions, such as study population and outcomes, they cannot become CER because they lack a control group.

The studies in 2011 by Viergever and Ghersi [35] and 2012 by Califf *et al*. [36] also reported the situation of retrospective registration. Retrospective registration (trial registration after enrollment of the first patient) may cause intended modification of the original protocols to obtain positive results [35]. The ICMJE and WHO declared that they require prospective registration of clinical trials before enrollment of the first patient. However, only 10.9% of the trials in our analysis of UMIN-CTR were registered (disclosure of trial information) before trial commencement (enrollment of first patient). This value remained low compared with reports from studies on ICTRP (26.0%) and ClinicalTrials.gov (48.0%).

With respect to publication of trial results, 90.0% of the trials in our analysis were unpublished. This may imply publication bias to some extent. However, almost all trials were registered as 'unpublished’ when they were first registered, and some registrants may forget to update registered trial information after they have published trial information. Hence, such trials remain 'unpublished’. In addition, some trials that are ongoing or submitting their results to medical journals were included in our analysis. Hence, the accurate prevalence of publication of these trials needs to be examined in more detail.

### Research question 3: Accessibility of trials registered in UMIN-CTR through ICTRP

The reason why study 'C000000060’ was not found in the search of ICTRP remains unclear. Our search strategy for ICTRP may not be sufficient to find all the studies imported from UMIN-CTR. However, it can be seen that, with the exception of this one trial, all 3,063 other clinical trials registered in UMIN-CTR during the 5-year period could be accessed by people around the world through ICTRP in the English language. Individuals can browse the 20-item dataset of these trials in ICTRP and visit the webpage for each trial in UMIN-CTR through a linked URL for more detailed information. UMIN-CTR is considered to contribute positively to the improvement of transparency and accessibility of clinical trials around the world.

### Research question 4: Accessibility of trials conducted in Japan (with at least one listed site in Japan) but registered abroad to Japanese people in the Japanese language

Our analysis showed that among the clinical trials conducted in Japan and registered in ClinicalTrials.gov from 2 June 2005 to 1 June 2010, only 8.7% were also registered in Japanese registries to be accessible in the Japanese language. A total of 1,286 clinical trials conducted in Japan were only registered in ClinicalTrials.gov in the English language. Considering the language difference, the accessibility of the information for those trials to Japanese individuals is being questioned. On the other hand, if a clinical trial is conducted in Japan and intended to be registered in UMIN-CTR, in accordance with the UMIN-CTR principle introduced in the Methods section, it must be registered not only in English but also in Japanese. The language problem in clinical trial registration has been discussed by other researchers [42]. We consider that providing information for clinical trials conducted in one country not only in English, but also in the first language of that country (if not English) has positive effects on fulfilling the ethical obligations of clinical trials, facilitating local people’s understanding of clinical trials, and promoting the development of clinical trial enterprises across that country.

When analyzing the type of funding source in the trials identified in our analysis, industry-funded trials were found to have a significantly lower prevalence (5.1%) of registration in Japanese registries than others-funded trials (19.0%) and industry + others-funded trials (14.8%) (Table 5). Industry-funded trials also accounted for 75.8% of the trials that were conducted in Japan but not registered in Japanese registries. Most industry-funded trials conducted in Japan are trials for new drug application and post-marketing clinical trials. At present, no legal document or ethical guideline in Japan requires the registration of such trials in Japanese registries. Even the ICMJE announcement requires only trial registration in any one of the registries acceptable to the ICMJE. Considering that it is not necessary to register a trial in more than one registry, many registrants may choose to only register trials in ClinicalTrials.gov because it is well-known around the world. Moreover, for global trials conducted simultaneously in Japan and the USA, registration in ClinicalTrials.gov is mandated by FDAAA 801 [30]. Hence, even if a trial also recruits patients from Japan, the trial information is likely to be only registered in ClinicalTrials.gov in the English language. However, we feel that exploration of how to improve the accessibility of such trials by Japanese people in the Japanese language is justifiable for the positive effects of first language registration as described above.

The prevalence of registration in Japanese registries among others-funded and industry + others-funded trials was not high. Most others-funded trials conducted in Japan are not trials for new drug application or post-marketing clinical trials. Although the Ethical Guidelines for Clinical Research issued by the MHLW have required the registration of such trials in Japanese registries since April 2009, its influence may not be well reflected in our analysis because we only included trials registered before 1 June 2010. The effects of the Ethical Guidelines for Clinical Research on Japanese clinical trial registration need to be observed in future studies.

### Limitations

Our research had some limitations. First, we did not include industry-funded trials or multinational trials not only conducted in Japan in research questions 1 and 2. Thus, our analyses do not reflect the characteristics of such trials. To investigate such trials, other clinical trial registries such as Japic-CTI (in which a large number of industry-funded trials are registered) and foreign registries (such as ClinicalTrials.gov) should be used.

Second, although we focused our analysis on academic clinical trials for research questions 1 and 2 by excluding industry-funded trials, this screening is merely based on the main funding source registered by the trial registrants. However, many clinical trials (especially large-scale trials) are funded by multiple sources and some clinical trials labeled as 'self-funded’ or 'foundation-funded’ may receive funds from industry. Hence, some industry-funded trials were inevitably included in our analyses for research questions 1 and 2. As Sawata and Tsutani [43] pointed out in their research, the problem of an unclear funding source is present in many Japanese clinical trials. Legislation or guidelines for the disclosure of real funding sources in clinical trials by the Japanese government and the corresponding improvement of the current clinical trial registration system are considered to be the solutions to this problem.

Third, we used ClinicalTrials.gov as a sample in the analysis for research question 4 to reveal the problem of accessibility of clinical trials conducted in Japan but registered abroad to Japanese people in the Japanese language. However, clinical trials conducted in Japan and registered in foreign registries other than ClinicalTrials.gov were not included in our analysis. This is only primary research, and more detailed studies on this topic are expected in the future.

## Conclusions

The present study is the first analysis of UMIN-CTR, which is the largest clinical trial registry in Japan, utilizing the information for registered clinical trials in the first 5 years since its establishment. We focused our research on Japan domestic, academic clinical trials in UMIN-CTR, and analyzed the registration trend and overall characteristics of those trials. The ICMJE announcements about clinical trial registration and the Ethical Guidelines for Clinical Research issued by the Japanese government are considered to have been positive factors in promoting clinical trial registration in UMIN-CTR. In regard to trial design, many trials adopted a single-arm design. Such trials may limit the overall quality of clinical trials and the development of CER in Japan. The reasons behind this phenomenon need to be explored in the future. Moreover, the problems of retrospective registration and possible low prevalence of publication of trial results pose big challenges for trial registration in UMIN-CTR.

We conducted a primary investigation on the accessibility of clinical trials registered in UMIN-CTR to people around the world through ICTRP, as well as the accessibility of clinical trials conducted in Japan (with at least one listed site in Japan) but registered abroad to Japanese people in the Japanese language. Almost all clinical trials registered in UMIN-CTR can be accessed by people around the world through ICTRP. On the other hand, many trials conducted in Japan but registered abroad cannot be accessed by Japanese people in the Japanese language. The aspect of how to improve the accessibility of such trials in the Japanese language (especially industry-funded trials) deserves further consideration.

## Abbreviations

CER: comparative effectiveness research; CONSORT: Consolidated Standards of Reporting Trials; EFPIA: European Federation of Pharmaceutical Industries and Associations; FDA: Food and Drug Administration; FDAAA: Food and Drug Administration Amendments Act; FDAMA: Food and Drug Administration Modernization Act; GCP: Good Clinical Practice; ICMJE: International Committee of Medical Journal Editors; ICTRP: International Clinical Trials Registry Platform; IDE: investigational device exemption; IFPMA: International Federation of Pharmaceutical Manufacturers & Associations; IND: Investigational New Drug application; Japic-CTI: Japan Pharmaceutical Information Center Clinical Trials Information; JMACCT: Japan Medical Association Center for Clinical Trials; JPMA: Japanese Pharmaceutical Manufacturers Association; JPRN: Japan Primary Registries Network; MEXT: Ministry of Education, Culture, Sports, Science and Technology; MHLW: Ministry of Health, Labour and Welfare; MHW: Ministry of Health and Welfare; NIH: National Institutes of Health; NIPH: National Institute of Public Health; PAL: Pharmaceutical Affairs Law; PhRMA: Pharmaceutical Research and Manufacturers of America; PMDA: Pharmaceuticals and Medical Devices Agency; RCT: randomized controlled trial; UMIN-CTR: University Hospital Medical Information Network Clinical Trials Registry; WHO: World Health Organization.

## Competing interests

All the authors declare that they have no competing interests.

## Authors’ contributions

HI, KT, and TK conceived and designed the study. MF and WT conducted the statistical analyses for all research questions. HI and TK were responsible for data acquisition. WT wrote the first draft of the manuscript. All authors read and approved the final manuscript.

## Supplementary Material

Additional file 1 **Title of data: Items of secondary concern.** Description of data: Results of analysis on items of secondary concern. Click here for file
