# Supplementary material for: Review of the registration of clinical trials in UMIN-CTR from 2 June 2005 to 1 June 2010 - focus on Japan domestic, academic clinical trials
Source: Trials. 2013 Oct 14;14:333. doi: 10.1186/1745-6215-14-333 (PMC4015268; doi:10.1186/1745-6215-14-333)
Supplement: Additional file 1 — Title of data: Items of secondary concern. Description of data: Results of analysis on items of secondary concern. [file 1745-6215-14-333-S1.doc]

| **Table S-1. Items of secondary concern** | | | | | |
| --- | --- | --- | --- | --- | --- |
| **Category** | | **No. of trials** | | **%** | |
| **1. Objective** | |  | |  | |
| Safety | | 168 | | 6.0 | |
| Efficacy | | 1,001 | | 36.0 | |
| Safety+Efficacy | | 1,396 | | 50.2 | |
| Bioequivalence | | 35 | | 1.3 | |
| Bioavailability | | 8 | | 0.3 | |
| Pharmacokinetics (PK) | | 36 | | 1.3 | |
| Pharmacodynamics (PD) | | 28 | | 1.0 | |
| PK+PD | | 10 | | 0.4 | |
| Others | | 97 | | 3.5 | |
| **2. Developmental phase** | |  | |  | |
| Phase I | | 118 | | 4.2 | |
| Phase I/II | | 141 | | 5.1 | |
| Phase II | | 569 | | 20.5 | |
| Phase II/III | | 52 | | 1.9 | |
| Phase III | | 211 | | 7.6 | |
| Phase IV | | 163 | | 5.9 | |
| Not applicable | | 553 | | 19.9 | |
| Missing data | | 972 | | 35.0 | |
| **3. Randomization unit** | |  | |  | |
| Individual | | 977 | | 35.2 | |
| Cluster | | 48 | | 1.7 | |
| Missing data | | 1,754 | | 63.1 | |
| **4. Stratification** | |  | |  | |
| Yes | | 466 | | 16.8 | |
| No | | 450 | | 16.2 | |
| Missing data | | 1,863 | | 67.0 | |
| **5. Dynamic allocation** | |  | |  | |
| Yes | | 419 | | 15.1 | |
| No | | 461 | | 16.6 | |
| Missing data | | 1,899 | | 68.3 | |
| **6. Institution consideration in allocation** | |  | |  | |
| Considered as an adjustment factor | | 227 | | 8.2 | |
| Considered as a block | | 131 | | 4.7 | |
| Not considered | | 443 | | 15.9 | |
| Missing data | | 1,978 | | 71.2 | |
|  | | | | | |
| **Table S-1. Continued** | | | | | |
| **Category** | | **No. of trials** | | **%** | |
| **7. Concealment** | |  | |  | |
| Central registration | | 621 | | 22.3 | |
| Numbered container method | | 176 | | 6.3 | |
| Pseudo-randomization | | 41 | | 1.5 | |
| No need to know | | 82 | | 3.0 | |
| Missing data | | 1,859 | | 66.9 | |
| **8. Blocking in allocation** | |  | |  | |
| Yes | | 274 | | 9.9 | |
| No | | 533 | | 19.2 | |
| Missing data | | 1,972 | | 86.5 | |
| **9. Purpose of intervention** | |  | |  | |
| Treatment | | 2,399 | | 86.3 | |
| Prevention | | 215 | | 7.7 | |
| Diagnosis | | 111 | | 4.0 | |
| Educational, counseling, training | | 54 | | 1.9 | |
| **10. Age-lower limit (months old)** | |  | |  | |
| [0,1)^*^ | | 20 | | 0.7 | |
| [1,6) | | 2 | | 0.1 | |
| [6,12) | | 7 | | 0.3 | |
| [12,72) | | 47 | | 1.7 | |
| [72,240) | | 333 | | 2.0 | |
| Older than 240 months | | 1,974 | | 71.0 | |
| Not applicable | | 396 | | 14.2 | |
| **11. Sex** | |  | |  | |
| Male | | 108 | | 3.9 | |
| Female | | 194 | | 7.0 | |
| Male+Female | | 2,477 | | 89.1 | |
| **12. Primary sponsor** | |  | |  | |
| University (university  hospital included) | | 1,579 | | 56.8 | |
| Hospital (university hospital not included) | | 313 | | 11.3 | |
| Research institute | 30 | | 1.1 | |  |
| Government | 5 | | 0.2 | |  |
| Others | 281 | | 30.7 | |  |

| **Table S-1. Continued** | | |
| --- | --- | --- |
| **Category** | **No. of trials** | **%** |
| **13. Funding source** |  |  |
| Government | 764 | 27.5 |
| Non-profit foundation | 308 | 11.1 |
| Outside Japan | 6 | 0.2 |
| Self funding | 1,479 | 53.2 |
| Others | 222 | 8.0 |

^*^[0,1) means from 0 month to 1 month but 1 month

not included. The same for the other labels in this

category.
